# Real-Life Evidence of Mepolizumab Treatment in Chronic Rhinosinusitis with Nasal Polyps: A Multicentric Study

**DOI:** 10.3390/jcm13123575

**Published:** 2024-06-18

**Authors:** Carlo Cavaliere, Antonella Loperfido, Andrea Ciofalo, Loreta Di Michele, Elona Begvarfaj, Gianluca Bellocchi, Marcella Bugani, Marco de Vincentiis, Antonio Greco, Stefano Millarelli, Michaela Plath, Eleonora Sculco, Simonetta Masieri

**Affiliations:** 1 Department of Sense Organs, Sapienza University, 00185 Rome, Italy; andrea.ciofalo@uniroma1.it (A.C.);; 2 Otolaryngology Unit, San Camillo Forlanini Hospital, 00152 Rome, Italy; aloperfido@scamilloforlanini.rm.it (A.L.);; 3 Department of Pulmonary Interstitial Diseases, San Camillo Forlanini Hospital, 00152 Rome, Italy; ldimichele@scamilloforlanini.rm.it; 4 Department of Otorhinolaryngology, Head and Neck Surgery, University Hospital Heidelberg, 69120 Heidelberg, Germany; michaelamaria.plath@icloud.com; 5 Department of Translational and Precision Medicine, Sapienza University, 00185 Rome, Italy; eleonora.sculco@uniroma1.it; 6 Department of Oral and Maxillofacial Sciences, Sapienza University, 00185 Rome, Italy

**Keywords:** Mepolizumab, chronic rhinosinusitis with nasal polyps, CRSwNP, monoclonal antibodies, mAb, biologics, IL-5

## Abstract

**Background**: The introduction of biological drugs in the management of chronic rhinosinusitis with nasal polyps (CRSwNP) is allowing new and increasingly promising therapeutic options. This manuscript aims to provide a multicenter trial in a real-life setting on Mepolizumab treatment for severe uncontrolled CRSwNP with or without comorbid asthma. **Methods**: A retrospective data analysis was jointly conducted at the Otolaryngology–Head and Neck Surgery departments of La Sapienza University and San Camillo Forlanini Hospital in Rome. Both institutions participated by sharing clinical information on patients with CRSwNP treated with Mepolizumab. Patients were evaluated before starting Mepolizumab, at six months and at twelve months from the first drug administration. During follow–up visits, patients underwent endoscopic evaluation, quality of life assessment, nasal symptoms assessment, and blood tests to monitor mainly neutrophils, basophils, eosinophils, and IgG, IgA, and IgE assay. **Results**: Twenty patients affected by CRSwNP and treated with Mepolizumab were enrolled (12 females and 8 males with a mean age of 63.7 years). Sixteen patients (80%) had concomitant asthma. During follow-up, a gradual improvement in nasal polyp score, quality of life and nasal symptoms, assessed by SNOT-22 and VAS and loss of smell measured by olfactory VAS, was found. Regarding blood tests, eosinophils decreased gradually, while other blood parameters showed no statistically significant changes. **Conclusions**: Mepolizumab has been shown to be effective in the therapeutic management of patients with CRSwNP. Further studies are needed to support our findings and better understand the underlying immune pathways to predict patients’ response to biological treatment in CRSwNP.

## 1. Introduction

Mepolizumab represents a humanized IgG1/kappa class monoclonal antibody (mAb) that acts on type 2 inflammation by selectively targeting human interleukin-5 (IL-5), blocking its association with the α chain of the IL-5 receptor complex (IL-5R) and thus inhibiting its subsequent activities [1]. In particular, IL-5 is implicated in the recruitment, differentiation, survival, and degranulation of eosinophils, which play a crucial role in airways inflammation [2]. Due to Mepolizumab’s high-affinity interaction, this mAb has a good safety and tolerability profile since it does not interfere with other inflammatory cytokines besides IL-5 [3].

According to the Global Initiative for Asthma (GINA) guidelines, Mepolizumab is recommended in patients over six years of age with severe eosinophilic asthma, uncontrolled despite maximum standard therapy [4]. Additionally, this mAb received Food and Drug Administration (FDA) and European Medicines Agency (EMA) approval for use in eosinophilic granulomatosis with polyangiitis (EGPA), hyper-eosinophilic syndrome (HES), and chronic rhinosinusitis with nasal polyps (CRSwNP) [5,6].

In Italy, Mepolizumab was approved by the Italian Agency of Drugs (AIFA) only in the last year, in March 2023, as an add-on therapy to the standard medical treatment with saline rinses and intranasal corticosteroids (INCS), for patients older than 18 years with severe CRSwNP, defined by extensive nasal polyps in both nasal cavities at nasal endoscopy (Nasal Polyp Score, NPS ≥ 5) and with a high impact of the disease on quality of life (Sino Nasal Outcome Test, SNOT-22 ≥ 50), for whom oral corticosteroid treatment and/or surgery do not provide adequate disease control [7].

More widely, European guidelines indicate biological treatment for patients with CRSwNP uncontrolled despite surgery and who have at least three of the following criteria: evidence of type 2 inflammation, need for systemic corticosteroids or contraindication to systemic steroids, significantly impaired quality of life, significant loss of smell, diagnosis of comorbid asthma [8].

Standard therapy for CRSwNP includes saline nasal irrigations and long-term INCS, short courses of oral corticosteroids for disease exacerbations, and the surgical option in patients in whom medical treatment is not sufficient. In particular, Functional Endoscopic Sinus Surgery (FESS) or non-functional Endoscopic Sinus Surgery (ESS), depending on the severity of the disease and number of previous surgeries, constitutes the gold standard approach in the surgical management of CRSwNP. However, the limitations of the standard pharmacological treatments are well documented, including possible side effects from prolonged oral corticosteroid use, such as diabetes, weight gain, facial swelling, infections, anxiety, insomnia, hyperactivity, mood swings, and reduced bone mineral density [9]. Similarly, surgery is not free from possible intra- or post-operative complications, and recurrences of nasal polyps after sinus surgery are frequent. In particular, recurrence of nasal polyposis with associated inadequate symptom control is a common occurrence in CRSwNP patients even after FESS and can be found in a large percentage of patients up to eighteen to twenty-four months. Specifically, factors potentially promoting CRSwNP recurrence after surgery are incomplete primary surgical approach in opening all paranasal sinuses, comorbid asthma and/or Widal disease (which corresponds to the clinical association of bronchial asthma, CRSwNP, and NSAID–ERD), allergy, occupational dust exposure, extent of disease at the time of surgery, and history of previous multiple surgeries. In fact, in a meta-analysis of over 43 clinical studies and 30,000 patients, Loftus et al. observed that the surgical revision rate in CRSwNP was 22.7% in patients with comorbid asthma and 27.2% in patients affected by NSAID–ERD compared to an 8% revision rate for patients without asthma [10]. For all these reasons, CRSwNP is still challenging to manage because of its tendency to recur [11]. New biologic treatments targeting the inflammation underlying CRSwNP are encouraging clinicians to manage more severe cases of CRSwNP that are not controlled by standard therapies [12,13]. They have also improved otolaryngologists’ diagnostic approach to CRSwNP, with increasing attention to the endo-typing of nasal polyposis [14]. Currently approved mAbs for CRSwNP include Mepolizumab (anti-IL5), Dupilumab (anti-IL4, IL13), and Omalizumab (anti-IgE) [15].

Regarding Mepolizumab, anti-IL5 therapy has been proven beneficial in treating severe and recalcitrant CRSwNP, with a reduction in nasal polyps’ dimensions and symptoms related to CRSwNP [16,17].

This study aims to report our real-life experience in managing CRSwNP patients with Mepolizumab, evaluating the disease control and efficacy of this biologic drug in the treatment of uncontrolled CRSwNP with or without comorbid asthma.

## 2. Materials and Methods

Retrospective data analysis was jointly performed at the Otolaryngology-Head and Neck Surgery departments of La Sapienza University–Umberto I Hospital and San Camillo Forlanini Hospital in Rome. Both institutions participated in this real-life study by sharing clinical information on patients with uncontrolled severe CRSwNP with or without comorbid asthma treated with Mepolizumab. Furthermore, since otolaryngologists have only recently been able to prescribe the drug, patients suffering from CRSwNP and asthma currently being treated with Mepolizumab on pneumological and immunological prescription were also enrolled.

In accordance with the AIFA guidelines, the biologic was administered through 100 mg subcutaneous injection administered once every four weeks as add-on therapy to INCS. The study population included all patients referred for treatment with Mepolizumab following the AIFA guidelines and the EPOS/EUFOREA update, and thus over 18 years of age with severe CRSwNP not controlled by standard therapies and with blood eosinophil counts > 150 cells/mL [7,18]. Exclusion criteria for treatment with Mepolizumab included patients under the age of 18, pregnancy, patients who did not consent to start biological therapy, patients being treated with other biological or immunosuppressive treatment for different reasons, cancer patients undergoing adjuvant therapy or who had been treated with radiotherapy or chemotherapy in the previous twelve months and patients on concomitant long-term steroid therapy for chronic autoimmune conditions. Furthermore, patients who had not signed their consent to share their clinical information were excluded from the study. Ethics committee approval was provided (Prot. N 411/CE Lazio1 19 April 2022), and informed consent regarding utilization of clinical data and privacy was obtained from all patients at the time of collection.

Patients were assessed at baseline (T0) before starting Mepolizumab and underwent a follow-up visit every six months: T1, 6 months of treatment; T2, 12 months of treatment.

Before starting the biological treatment, each patient underwent a complete medical history, including sex, age, smoking habit, presence of concomitant asthma, allergies and previous surgery for treatment of CRSwNP. In addition, a complete blood count and immunoglobulin (Ig)G, IgA, and IgE assay were required for each patient.

Furthermore, to objectively detect the presence of CRSwNP and the extent of nasal polyposis, each patient underwent a complete ENT physical examination, including nasal endoscopy. NPS represents the staging system routinely used in our clinical practice to quantify disease severity at endoscopic evaluation [19]. Each patient also required a sinus computed tomography (CT) scan to evaluate sinonasal disease radiologically.

Symptom severity and health-related QoL were assessed through the Visual Analog Scale (VAS) and SNOT-22. VAS evaluates the intensity of total symptoms, measured with a scale of values ranging from 0 (no discomfort) to 10 (maximum discomfort). Evaluated symptoms are those typically associated with chronic rhinosinusitis and include nasal obstruction, nasal discharge, impaired smell, post-nasal drip, and headache [20]. A smell assessment was specifically performed using the olfactory VAS.

The SNOT-22 questionnaire consists of 22 personalized questions concerning health-related QoL and symptom severity in patients with inflammatory diseases of the nose and paranasal sinuses. The outcomes assessed are classified into two groups: physical symptoms (items 1–12), which include symptoms related to the nasal district (items 1–8), ear symptoms (items 9–11), and facial symptoms (item 12); and QoL and psychological state (items 13–22) which specifically assess patients’ sleep (items 13–16) and mental sensations (items 17–22). The total result score can vary from 0 to 110 [21]. Specifically, the sum of the scores of each SNOT-22 question is equal to the severity of nasal symptoms: a score greater than 50 corresponds to severe symptoms, between 20 and 49 to moderate symptoms, and less than 20 to mild symptoms [22]. Therefore, a score above 50 is indicative of severe CRSwNP. Then, during follow-up visits, patients underwent endoscopic evaluation, assessment of symptom severity and QoL, evaluation of sense of smell, and blood tests to monitor mainly neutrophils, basophils, and eosinophils, as well as an IgG, IgA, and IgE assay.

Data from the enrolled cases were analyzed using the Friedman test for repeated measures and the Bonferroni test as post hoc; data are expressed as mean (SD). All analyses were conducted with STATA v13 software (StataCorp Release 13. College Station, TX, USA).

## 3. Results

The study collected twenty patients affected by CRSwNP and treated with Mepolizumab. Twelve were females, and eight were males, showing a female prevalence. The mean age was 63.7 years (range from 47 to 86 years). Sixteen patients (80%) had concomitant asthma. Six patients (30%) were cigarette smokers, and 4 patients were previous smokers, all of whom stopped had smoking for over 10 years. Eleven patients (55%) were allergic; of these, only one was to perennial allergens (*Dermatophagoides Pteronissunus*), and the remainder were allergic to *Parietaria officinalis*, grasses, and cypress. Seventeen patients (85% of the entire cohort) underwent at least one FESS before starting Mepolizumab. The total mean was 1.45 surgeries per subject (range from 1.0 to 3.0 surgeries). Table 1 summarizes the baseline parameters.

During follow-up, a gradual improvement in NPS, QoL, nasal symptoms assessed by SNOT-22 and VAS, and loss of smell measured by olfactory VAS was found.

Specifically, for each clinical parameter that showed a statistically significant change during Mepolizumab therapy, we have included the corresponding figure. For parameters that did not exhibit a statistically significant change during the therapy, we describe the trend in the text without including specific figures.

**Figure 1:**
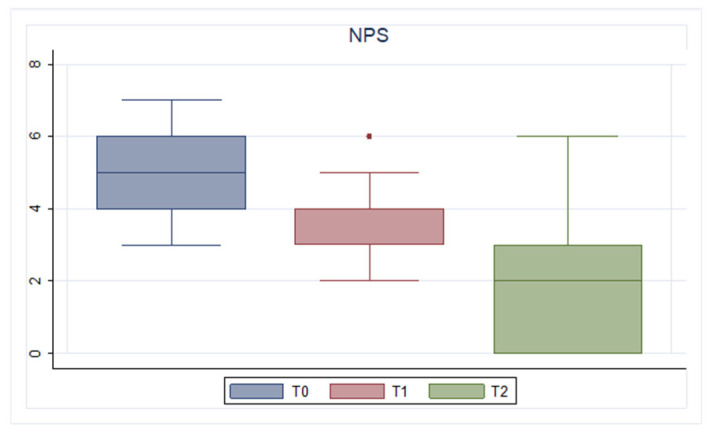
NPS trend. T0: baseline; T1: 6 months of treatment; T2 12 months of treatment.

In particular, a gradual improvement in NPS was found in the extension of nasal polyps, as shown in Figure 1. Specifically, the mean value before starting Mepolizumab was 5.11 (1.05). After six months of treatment, the score improved to 3.65 (1.04), and after one year there was further improvement, reaching a mean value of 1.89 (1.73) (*p* < 0.001).

**Figure 2:**
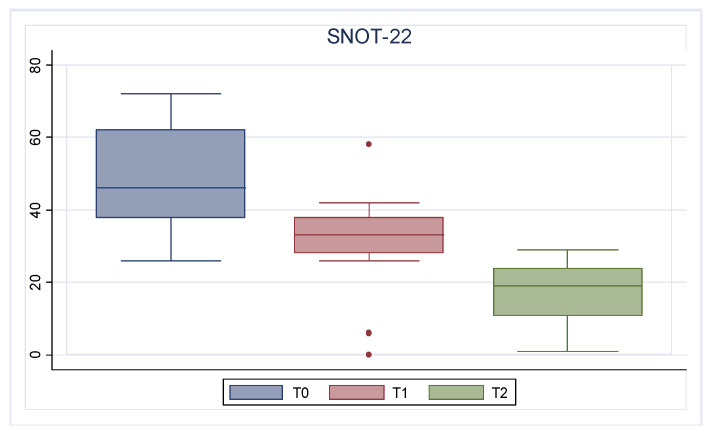
SNOT-22 trend. T0: baseline; T1: 6 months of treatment; T2 12 months of treatment.

**Figure 3:**
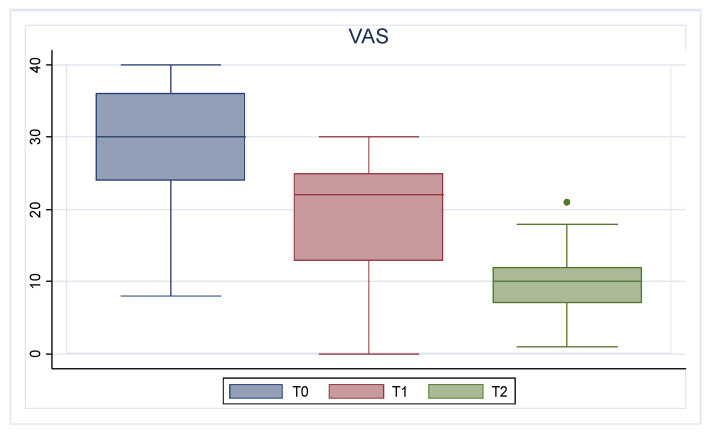
VAS trend. T0: baseline; T1: 6 months of treatment; T2 12 months of treatment.

Focusing on symptom severity and QoL, the mean SNOT-22 value at baseline was 48.32 (13.20); after six months, the value decreased to 31.89 (12.37), and after twelve months, it was 16.59 (8.49) (*p* < 0.001). Concerning VAS, the mean value improved from 29.37 (8.17) at T0 to 18.79 (8.16) at T1 and 10.29 (6.01) at T2 (*p* < 0.001). Figure 2 and Figure 3 describe the trends of SNOT-22 and VAS, respectively.

**Figure 4:**
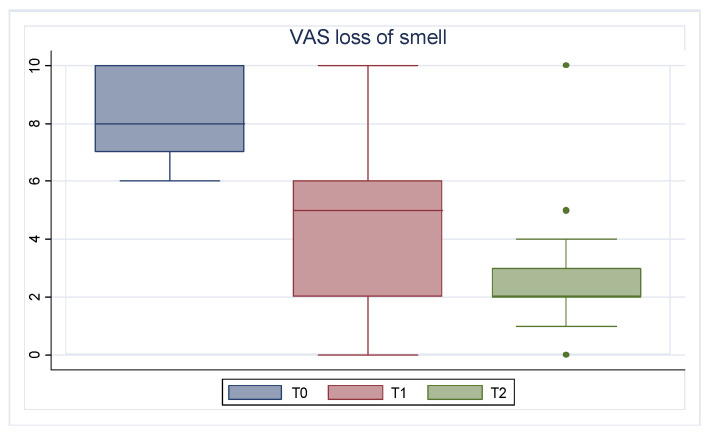
Olfactory VAS trend. T0: baseline; T1: 6 months of treatment; T2 12 months of treatment.

Regarding loss of smell, the olfactory VAS mean value improved from 8.47 (1.31) at baseline to 4.05 (2.59) after six months. This improvement was confirmed at one year of therapy, reaching a mean value equal to 2.71 (1.38) (*p* < 0.001). The improvement of the olfactory VAS is shown in Figure 4.

**Figure 5:**
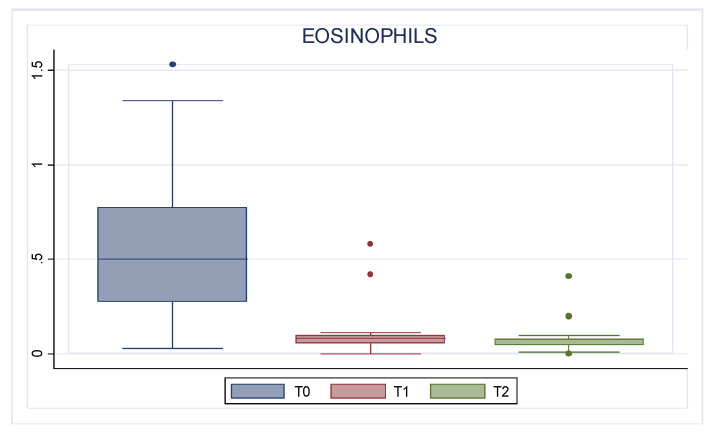
Absolute eosinophil count trend. T0: baseline; T1: 6 months of treatment; T2 12 months of treatment.

Concerning blood tests, eosinophils decreased gradually. The mean absolute value of 0.58 cells × 10^9^/L (0.42) before treatment decreased to 0.12 cells × 10^9^/L (0.49) at T1 to 0.09 cells × 10^9^/L (0.11) at T2 (*p* < 0.001). Figure 5 reports the absolute eosinophil count trend.

Neutrophils and basophils remained within the normal range without demonstrating significant changes in their trend. Specifically, regarding neutrophils, the mean value before treatment was 3.62 × cells 10^9^/L (1.18); at T1, it was 4.12 × cells 10^9^/L (1.94), and at T2, the mean value was 3.37 × cells 10^9^/L (1.5) (*p* = 0.558). Concerning basophils, at T0, the mean value was equal to 0.07 cells × 10^9^/L (0.07); at T1, the mean value was 0.04 cells × 10^9^/L (0.04); at T2, the mean value was 0.05 cells × 10^9^/L (0.05) (*p* = 0.358).

Patients’ immunity was further assessed by measuring total serum IgG, IgA, and IgE trends during treatment. Total IgG remained in the normal range (corresponding to 700–1600 mg/dL [23]), going from a value of 1060.73 mg/dL (164.84) at baseline to a value of 1075.40 mg/dL (155.72) at T1 to a value of 1105.70 (109.41) at T2 (*p* = 0.452). IgA also remained in the normal range (corresponding to 70–400 mg/dL [23]) and showed no significant changes in the trend, as the mean value at T0 was 250.91 mg/dL (75.85), at T1 was 261.40 mg/dL (72.92) and at T2 was 272.60 mg/dL (62.43) (*p* = 0.717). Concerning total IgE, the mean value at T0 corresponded to 342.48 UI/mL (508.04); after six months of therapy, IgE decreased to a mean value of 153.32 UI/mL (130.47), and after twelve months of treatment, IgE increased to a mean value of 226.73 UI/mL (401.19) (*p* = 0.905). Assuming that the normal range of total IgE in adults corresponds to 0–100 UI/mL, these immunoglobulins remained above the standard value despite Mepolizumab, and we found no statistically significant variability in IgE levels during treatment.

Regarding adverse effects, Mepolizumab was well tolerated and no serious adverse reactions were reported. Only one patient complained of transient back pain after the second injection, which resolved spontaneously after a few weeks.

## 4. Discussion

CRSwNP represents a chronic inflammatory disease of the nose and paranasal sinuses with an estimated prevalence between 2.1 and 4.4% in the general population in Europe and approximately 1.1% in the USA [24]. The pathological features of CRSwNP are complex, as they involve chronic inflammation, epithelial damage and repair reactions, eosinophil and macrophage infiltration, and tissue remodeling [25]. This chronic inflammatory condition is often severe and has a significant negative impact on patients’ sleep and physical and mental status, resulting in impaired health-related quality of life (QoL) comparable to that of other debilitating chronic diseases, such as chronic obstructive pulmonary disease (COPD), diabetes, asthma, and congestive heart failure [26]. CRSwNP in the Caucasian population is typically eosinophilic, driven by type 2 cytokines such as interleukin (IL)-4, IL-5, and IL-13 [27], resulting in symptoms of nasal obstruction, nasal discharge, facial pain or facial pressure and loss of smell for at least twelve weeks [28]. Specifically, the type 2 inflammatory pathway is associated with inflammatory diseases that include not only CRSwNP but also asthma, non-steroidal anti-inflammatory drug exacerbated respiratory disease (NSAID–ERD), eosinophilic COPD, allergic rhinitis, atopic dermatitis, chronic prurigo, chronic urticaria and eosinophilic esophagitis [29,30]. As a consequence of this shared pathway, patients affected by CRSwNP often suffer simultaneously from one of these comorbid conditions, particularly asthma and/or NSAID–ERD, resulting in an increased disease burden [31].

It has been widely demonstrated in the literature that IL-5 represents a cytokine with a significant role in the pathogenesis of inflammatory endotype 2 and CRSwNP [32]. Specifically, this inflammation biomarker is essential for both eosinophil cell differentiation, including the proliferation of eosinophil precursors in the bone marrow, and for mature eosinophils, promoting activation, survival, and migration of these cells into peripheral tissues [33]. The main cellular sources of IL-5 comprise Th2 lymphocytes, mast cells, group 2 innate lymphoid cells (ILC-2), CD34+ progenitor cells, and invariant natural killer T cells. In addition, this interleukin can also be secreted by eosinophils through an autocrine/paracrine mechanism [34]. IL-5 communicates through receptors for IL-5 (IL-5R), formed by α and β transmembrane chains; however, IL-5 can also bind to a soluble IL-5 receptor alpha (IL-5Rα) chain form, inhibiting IL-5R signal. Therefore, the eosinophils’ responsiveness to IL-5 depends on the level of expression of soluble and transmembrane IL-5Rα, which in turn depends on the eosinophils’ maturation, activation state, and localization [35]. Tissue-resident eosinophils selectively show a lower transmembrane IL-5Rα expression, demonstrating less sensitivity to anti-IL5 than circulating eosinophils [36]. For some authors, this may explain why anti-IL-5 therapy in patients affected by eosinophilic diseases, such as severe eosinophilic asthma, causes a greater decrease in circulating eosinophils than in tissue-resident eosinophils [37,38]. Three anti-IL-5 biologics directed against this cytokine or its receptor have been approved for clinical use: Mepolizumab, Reslizumab, and Benralizumab. Specifically, Mepolizumab and Reslizumab directly target IL-5 and prevent the interaction between IL-5 and eosinophils, resulting in reduced production and survival of eosinophilic cells. In contrast, Benralizumab directly targets IL-5Rα, resulting in cellular cytotoxicity and depletion of eosinophils and other IL-5Rα-bearing cells [39,40]. All three mAbs have been approved for the treatment of severe refractory eosinophilic asthma while, for CRSwNP, the only anti-IL5 biologic currently available is Mepolizumab.

In 2011, a first randomized, double-blind, placebo-controlled trial was published regarding the use of Mepolizumab in the therapeutic management of CRSwNP. The trial included twenty patients with uncontrolled severe CRSwNP treated with two intravenous injections of 750 mg Mepolizumab at a 4-week interval and ten patients with uncontrolled severe CRSwNP treated with a placebo following an equal protocol. The NPS, blood eosinophil count, serum IL-5Ra, and eosinophil cationic protein (ECP) levels in patients on Mepolizumab therapy were significantly reduced from baseline compared with the placebo group. In contrast, reductions in SNOT-22 score, CT Lund Mackay score, nasal discharge score, and loss-of-smell symptom score were not significantly different between the Mepolizumab and placebo groups [41]. In 2017, Bachert et al. published the next randomized, double-blind, placebo-controlled trial regarding the therapeutic role of Mepolizumab in patients affected by CRSwNP. One hundred and seven patients were included in the study and randomly divided into the Mepolizumab group (fifty-four subjects) and the placebo group (fifty-three subjects). The collected patients had severe uncontrolled CRSwNP, defined by VAS greater than seven and NPS greater than or equal to 3 in one nasal fossa and greater than or equal to 2 in the other nasal fossa. Patients in the Mepolizumab group received 750 mg of the biologic drug by intravenous injection every four weeks, taking a total of six doses. As a result, NPS, SNOT-22, and VAS scores significantly decreased in patients treated with Mepolizumab compared to the placebo group. Furthermore, at the week 25 assessment, a significantly greater percentage of patients in the Mepolizumab group compared to the placebo group no longer required sinonasal surgery for CRSwNP. Additionally, at the week 25 assessment, a decrease in blood eosinophil counts was observed in the Mepolizumab group from 500 eosinophil cells/mL at baseline to 50 eosinophil cells/mL; in contrast, this decrease was not observed in the placebo group. This trial is critical to confirm the potential of Mepolizumab in improving QoL and symptoms and reducing the burden associated with surgery in patients with severe CRSwNP [42]. These results were confirmed by the SYNAPSE study, consisting of a randomized, double-blind, placebo-controlled phase 3 trial. Subcutaneous administration of 100 mg Mepolizumab once every four weeks resulted in reductions from baseline in SNOT-22 and NPS, improvement in VAS nasal obstruction score, and further confirmed the reduced need for a surgical approach during the 52-week treatment period [16]. In an additional clinical trial, the authors analyzed the SYNAPSE study to define whether the clinical benefits of Mepolizumab could be preserved after discontinuation of 52 weeks of biologic treatment over a 24-week follow-up period. Twenty-four weeks after treatment discontinuation, the authors found that the observed clinical improvements, particularly the risk of sinus surgery and the volume of nasal polyps, remained essentially decreased from baseline in the Mepolizumab-treated patients compared with placebo, while blood eosinophils returned to pre-treatment levels [43]. Chupp et al. demonstrated that Mepolizumab can reduce the need and use of systemic corticosteroids in patients affected by CRSwNP. Specifically, the authors noted that Mepolizumab enables the reduction of systemic corticosteroids use in CRSwNP patients with a wide variety of clinical features, including different number of previous surgeries, a range of blood counts of eosinophils at baseline, and subjects with and without NSAID–ERD or asthma comorbidities. Finally, it was observed, through nasal cytological sampling, how treatment with Mepolizumab significantly reduces the expression of vascular endothelial growth factor (VEGF) and its receptors (VEGFR1, VEGFR2) after 12 weeks of therapy, normalizing the expression of VEGFR1 and VEGFR2 compared to healthy controls [44,45].

Real-life experiences demonstrating the effectiveness of Mepolizumab in the therapeutic management of patients with CRSwNP are increasing in the literature, in line with our results showing improvement in NPS, QoL, and nasal symptoms as measured by VAS, SNOT-22, and olfactory VAS. In this regard, Gallo et al. verified the efficacy of Mepolizumab on sinonasal aspects in forty-three patients with severe asthma with concomitant CRSwNP, finding a significant improvement in clinical and endoscopic aspects after twelve months of treatment, with a reduction in symptom severity (assessed by SNOT-22), nasal polyps’ volume (measured by NPS), and blood eosinophils [46].

Domínguez-Sosa et al. evaluated in a real-life setting the efficacy of Mepolizumab in fifty-five patients with severe CRSwNP by assessing several clinical parameters, including NPS, VAS, SNOT-22, asthma control test (ACT) score, exhaled fractional nitric oxide (FeNO), blood eosinophils and prednisone intake, at baseline and after six months. They found significant efficacy of Mepolizumab in reducing nasal symptoms, polyp scores, blood eosinophils, and systemic corticosteroid use. In conclusion, the authors affirmed the ability of this biologic to improve health-related QoL in these patients, regardless of whether they had asthma or NSAID–ERD disease as a comorbidity [47].

By contrast, some real-life studies have shown some disagreements on Mepolizumab’s ability to reduce the extent of nasal polyps [48,49,50].

The most interesting data we observed in our study demonstrate that Mepolizumab was very effective in restoring the sense of smell, as demonstrated by improved olfactory VAS. Our results are in line with the post hoc analysis of the SYNAPSE study, in which Mullol et al. emphasized the ability of Mepolizumab to improve olfaction in patients with severe refractory CRSwNP, as assessed by SNOT-22 sense of smell/taste item score and loss of smell VAS score [51].

While basophils and neutrophils showed no significant changes, eosinophils presented a reduction from baseline to post-treatment, in line with the results of other authors [52,53] and in agreement with the anti-IL5 mAb mechanism of action [54]. Regarding the impact of Mepolizumab on immunoglobulins, we found no significant changes in any of the Ig classes. Other authors also reported in their studies that Mepolizumab did not significantly change Ig levels in the blood. In particular, Contoli et al. compared the effects of two anti-IL5 biological treatments, specifically Mepolizumab and Benralizumab, on blood IgE levels in severe eosinophilic atopic asthmatic patients. The authors found that, while Benralizumab significantly reduced total blood IgE levels in patients treated with this mAb, Mepolizumab did not significantly change total blood IgE levels in patients receiving this biologic. Interestingly, the authors demonstrated that, in patients treated with Benralizumab, the reduction in total blood IgE levels was correlated with the decrease in blood basophils. In contrast, Mepolizumab demonstrated a marginal effect on basophils, consistent with our reported experience. The authors conclude that these results are due to the specific mechanisms of action of the different anti-IL5 treatments evaluated [55]. In addition, in a recent clinical trial of Mepolizumab treatment in aspirin-exacerbated respiratory disease, the authors found that there was no difference in IgE and IgG4 levels in patients taking or not taking this biologic drug [56].

To the very best of our knowledge, this is the first study in a real-life setting to evaluate the impact of Mepolizumab on several blood parameters, including IgE, IgG, and IgA, in patients being treated for CRswNP. However, there are several limitations to our series, which should be considered. First is the design of our study, as it is a retrospective analysis; secondly, the number of patients, being small, is not adequate to provide any conclusive evidence about changes in Ig expression, although our cohort is valid when compared with other published series [57]; furthermore, the duration of the follow-up period is limited to one year of treatment. More studies are required to support our findings and better understand the underlying immune mechanisms.

## 5. Conclusions

According to our preliminary findings regarding Mepolizumab, this mAb has been shown to be effective in the therapeutic management of patients with CRSwNP, promoting reduction of nasal polyps, decrease in blood eosinophils, improvement of disease-related symptoms, particularly sense of smell, and improvement of QoL. Further studies in a larger number of patients and a more extended follow-up period are needed to support our findings and better understand the underlying immune pathways to predict patients’ response to biological treatment in CRSwNP.

## Figures and Tables

**Table 1:** Patients’ baseline features.

Characteristic	N	Mean	SD
Patients	20	-	-
Male (%)/Female (%)	8 (40)/12(60)	-	-
Age (years)	-	63.7	11.95
Smoker (%)	6 (30%)	-	-
Asthma (%)	16 (80%)	-	-
Atopy (%)	11 (55%)	-	-
NPS	-	5.11	1.05
SNOT22	-	48.32	13.20
VAS	-	29.37	8.17
Olfactory VAS	-	8.47	1.31
Previous surgery (n; %)	17 (85%)	-	-
Number of surgeries (min-max; mean)	1–3	1.45	-
IgG (mg/dL)	-	1060.73	164.84
IgA (mg/dL)	-	250.91	75.85
IgE (UI/mL)	-	342.48	508.04
BASOPHILS (cells × 10^9^/L)	-	0.07	0.06
NEUTROPHILS (cells × 10^9^/L)	-	3.62	1.18
EOSINOPHILS (cells × 10^9^/L)	-	0.58	0.42

Legend. N: number; SD: standard deviation; NPS: Nasal Polyp Score; SNOT22: Sino Nasal Outcome Test 22; VAS: Visual Analog Scale; min: minimum; max: maximum.

## Data Availability

The data presented in this study are available on request from the corresponding author. The data are not publicly available due to privacy.
